# Atlanta Roentgen Ray Association, Regular Monthly Meeting, April 5, 1915

**Published:** 1915-04

**Authors:** 


### ATLANTA ROENTGEN RAY ASSOCIATION.

### Regular Monthly Meeting April 5, 1915.

Dr. DeLos L. Hill presented a Reading of Dental Radiographs. These embraced plates that showed the normal anatomy of the jaws and skull, the pathology of Aveolar Abscess, Necrosis, Maxillary Sinusitis and Root Fillings. The films were thrown upon a screen by a good lantern and were sufficiently enlarged to be seen easily and to have the various fundamental points demonstrated. The invasion and destruction of bone in abscess and necrosis was clearly shown and the methods of treatment required by the conditions were indicated.

In Root Fillings, the problems that have long confronted even the best of operators as to whether the root was exactly and completely filled were made clear. When there is incomplete filling, abscess will occur and where there has been over filling, there will be a rarefying osteitis as a result of the pressure of foreign substance. This is well known, of course, but hitherto, one has had to wait and see what happened after he had done his best to make the work perfect. Now he can observe the filling practically as he goes along and be certain what he has done.

The results of the accident of breaking an instrument off in a root canal were portrayed and the best way to remove it was indicated.

Impacted wisdom teeth were exactly located and this always great problem was simplified at least to the extent that the dentist could see in advance just what he had to do and prepare for it.

The conclusion was that one could hardly afford to practice without the aid of the X-Ray.

Dr. Gillespie Enloe said that the demonstration was complete and covered everything one could think of in its particular relation. There was no more guess work nor incertitude that even the most skillful and experienced man has had to feel in the matter of root surgery. He could know his work was well done and he could not only reassure his patient upon that point, but he could advise a new patient with confidence as what was needed.

Dr. J. S. Derr said that in through and through skiagraphs the abscess showed light instead of a shadow as in Hill's pictures and he asked why that was. Hill had mentioned a "fluorescence" that appeared in the film itself where there was no pus. This was new to him.

Hill said that he did not know why there was the difference Derr mentioned. The abscesses always showed dark in the work he had been doing.

Dr. J. S. Derr read a paper upon his "Impressions of the Fifteenth Annual Meeting of the American Roentgen Ray Society" in Cleveland. He also showed many films and plates some of them dental and others general in character.

Dr. Cosby Swanson read a paper on "X-Ray in the Treatment of External Diseases."

He showed among other things a penetrameter that enables him to determine just the character of the ray he wants to use in various cases.

Dr. Miller B. Hutchins said that he was the first man in the South and among the earliest anywhere to use the Roentgen Ray in treatment. His instruments and methods were very crude at first and he usually got a little more than the erythema dose, himself. He next bought a "not too powerful" machine some 13 years ago and with certain modifications he uses it now. He has burned only one patient and he did that purposely because of sarcoma of the cheek. The result justified him in this instance. He now uses the fractional dose method and has varying results. Among other things he has discarded attempting to remove superfluous hairs from a woman's face by the Ray. Scars result. In Fungating type of epitheleoma, he curettes everything away he can reach and then uses the Ray or caustic potash. In this condition they are of about equal value. As to penetration one had better use a hard or medium hard tube. The responsiveness of the skin decreases more rapidly than that of the deeper tissues and since the hard rays burn less they can more readily get through the skin and work upon the subcutaneous tissues than could the soft ones. In general, very few people have the time and money to take X-Ray treatment. They get a little better and cease to come. That is the one great reason why more has not been done with it. His own work has been limited to malignant cases where there was not room for operation.

As to protection of operators he has noted the fatiguing effect of the static atmosphere aside from burns. He uses aluminum and lead screens for burn protection and has a small cylinder of paper to assist him in directing the rays to small areas. The kerato-epithelioma on operator's hands will yield to radium or the high gamma ray. It is to be remembered that the live part of a cancer is not in the middle where it shows the most, but at border. It may be only stimulated to do greater destruction if the rays are directed solely at the center.

Dr. Derr said that he agrees with Swanson in advising the use of every instrument of precision to be had. Ray "tuning" can be approximated with experience, but it is better measured. He cured one case of Telangiectasis at the elbow with three light exposures of one half pastile strength. As to filters he uses leather and 3 m.m. of aluminum and several layers of cloth to get free of the soft rays. In this way tumors of the abdomen can be cured without skin troubles.

Dr. B. E. Sales showed some interesting dental plates along the line of Dr. Hill's. They were finely executed and confirmatory of Hill's statements.

Dr. G. M. Niles showed a plate of cancer of the stomach taken last December. It revealed a condition that might have been successfully treated surgically at that time and he so advised the man. Operation was declined, however, and the man recently died.

---

It is worth remembering that abduction is of great value in the prevention and in the treatment of stiffness of the shoulder after trauma.—American Journal of Surgery.

---

In cases of fracture of the base of the skull, operation is not indicated unless the cerebral symptoms are persistent or progressive.—American Journal of Surgery.

---

Wire sutures are unnecessary and undesirable in operating upon the fractured patella or olecranon. Kangaroo tendon, which is slowly absorbed, is strong enough.—American Journal of Surgery.

---

After injury to an extremeity localized tenderness or extensive ecchymosis is each sufficiently suggestive of a fracture to make an x-ray examination desirable, even though all other signs are absent.—American Journal of Surgery.

---

A "pathological fracture" is usually due to a bone tumor (sarcoma, carcinoma, myeloma), cyst or gumma. An expert interpretation of a radiograph will best serve to distinguish these. The Wassermann reaction, it must be remembered, is sometimes negative in tertiary syphilis.—American Journal of Surgery.

